# Automatic method of analysis and measurement of additional parameters of corneal deformation in the Corvis tonometer

**DOI:** 10.1186/1475-925X-13-150

**Published:** 2014-11-19

**Authors:** Robert Koprowski

**Affiliations:** Department of Biomedical Computer Systems, University of Silesia, Faculty of Computer Science and Materials Science, Institute of Computer Science, ul. Będzińska 39, Sosnowiec, 41-200 Poland

**Keywords:** Tonometer, Cornea, Eye, Biomechanics, Corvis ST, Image processing, Scheimpflug camera, Corneal deformation

## Abstract

**Introduction:**

The method for measuring intraocular pressure using the Corvis tonometer provides a sequence of images of corneal deformation. Deformations of the cornea are recorded using the ultra-high-speed Scheimpflug camera. This paper presents a new and reproducible method of analysis of corneal deformation images that allows for automatic measurements of new features, namely new three parameters unavailable in the original software.

**Material and method:**

The images subjected to processing had a resolution of 200 × 576 × 140 pixels. They were acquired from the Corvis tonometer and simulation. In total 14000 2D images were analysed. The image analysis method proposed by the author automatically detects the edge of the cornea and sclera fragments. For this purpose, new methods of image analysis and processing proposed by the author as well as those well-known, such as Canny filter, binarization, median filtering etc., have been used. The presented algorithms were implemented in Matlab (version 7.11.0.584 - R2010b) with Image Processing toolbox (version 7.1 -R2010b) using both known algorithms for image analysis and processing and those proposed by the author.

**Results:**

Owing to the proposed algorithm it is possible to determine three parameters: (1) the degree of the corneal reaction relative to the static position; (2) the corneal length changes; (3) the ratio of amplitude changes to the corneal deformation length. The corneal reaction is smaller by about 30.40% compared to its static position. The change in the corneal length during deformation is very small, approximately 1% of its original length. Parameter (3) enables to determine the applanation points with a correlation of 92% compared to the conventional method for calculating corneal flattening areas. The proposed algorithm provides reproducible results fully automatically within a few seconds/per patient using Core i7 processor.

**Conclusions:**

Using the proposed algorithm, it is possible to measure new, additional parameters of corneal deformation, which are not available in the original software. The presented analysis method provides three new parameters of the corneal reaction. Detailed clinical studies based on this method will be presented in subsequent papers.

## Introduction

**Figure 1:**
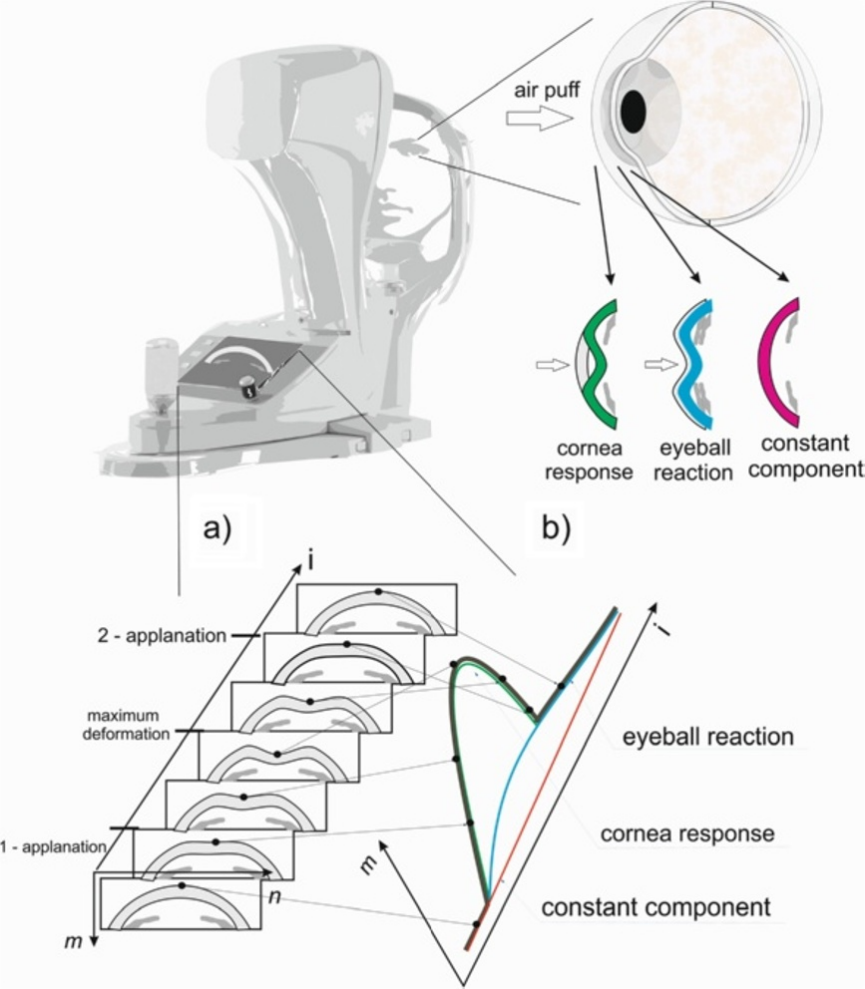
**Measurement method with the use of the Corvis tonometer and the obtained results of a sequence of corneal deformation images.** The cornea is subjected to an air puff in the Corvis tonometer. Consequently, every 230 μs an image of the corneal deformation in the line arranged on the main axis is obtained. Particular important deformation phases are shown on the left in the image **a)**. Reactions of the cornea and the eyeball are shown on the right in the image **b)**.

Current advances in technology enable to measure intraocular pressure using a number of methods. These are the methods of non-contact and impression applanation tonometry [1, 2]. One such type is the Corvis tonometer which allows for the quantitative and qualitative (visual) assessment of biomechanical properties of the cornea. Based on a sequence of images of corneal deformation, the tonometer measures corneal thickness, deformation amplitude, applanation length, corneal deformation speed and intraocular pressure [3]. A schematic sequence of corneal deformation images and selected parameters are shown in Figure 1. In recent years, these parameters have been the subject of many papers and comparisons both among themselves as well as among other types of tonometers [4–47].

For example, there is a group of publications related to the analysis of corneal biomechanical parameters for the Ocular Response Analyzer (ORA) [4–7] and the impact of the patient’s age [8–12], glaucoma [13–18] and wound healing [19] on the results obtained. Also predictive numerical simulation of corneal biomechanical parameters [20] and intraocular pressure measured in vivo [21] have been analysed. A significant number of papers is devoted to corneal hysteresis and its association with glaucoma damage [22], pachymetry [23–25] or hysteresis measured in children and healthy patients using the Reichert ocular response analyzer [26, 27]. Corneal hysteresis was also analysed for porcine eyes or open-angle glaucoma [28–30]. A significant part of the papers deals with the relationship between keratoconus and its biomechanical properties [31–33]. The situation is similar to assisted laser in situ keratomileusis [34, 35], or the ultrastructure of the corneal stroma [36]. Therefore corneal biomechanical properties [37–41] are measured in various ways with different types of tonometers [42, 43] for various types of diseases [44, 47].

In the case of the Corvis tonometer, the comparative analysis carried out in the literature (e.g. Smedowski el at [3]) applies only to the parameters available in the device (intraocular pressure, pachymetry, applanation 1 time, applanation 1, length, applanation 1 velocity, applanation 2 time, applanation 2 length, applanation 2 velocity, highest concavity time, peak distance, radius, deformation amplitude maximum). The corneal deformation image analysis enables to determine a significantly larger number of interesting parameters than those available in the original software [48–50]. Few papers have been published so far in this area. These include the papers of Tejwani et al. [51] and Koprowski et al. [52–54]. The first one ( [51]) refers to spectral analysis and comparison of the ORA with the Corvis tonometer. The second and third papers ( [52, 54]) present the analysis of new features (e.g. reaction of the eyeball) obtained from the image analysis method proposed by the authors. In this paper, a new analysis method of the cornea images acquired from the Corvis tonometer is presented. It allows for automatic measurements of new, not yet published, features of the cornea - unavailable in the original software. These three new automatically designated parameters are: the degree of the corneal reaction relative to the static position, the corneal length changes, the ratio of amplitude changes to the corneal deformation length. They are described in detail in the following sections.

## Material

As part of the paper, the correctness of the algorithm operation was tested on sequences of corneal deformation images obtained every 230 μs with a fixed *M* × *N* × *I* resolution of 200 × 576 × 140 pixels. The data of about 100 eyes were obtained from real data (open-access medical image repositories) and simulation data from the Corvis tonometer. Simulation data are derived from a corneal deformation generator, specially designed for this purpose, which provides any number of corneal deformations without patient’s participation. There were 140 2D images in each measurement, which in total gave 14000 2D images for analysis. The analysis presented in the following part of the paper enabled to enter image sequences in the source recording format *.cst or as a file *.avi.

## Method

The new method of data analysis consists of two stages. (1) Image pre-processing- the data from the Corvis tonometer was subjected to analysis which enabled reconstruction of the cornea shape changes and separation of the eyeball reaction and corneal deformation; (2) The corneal reaction was analysed, which resulted in three new parameters.

The presented algorithms were implemented in Matlab (Version 7.11.0.584 - R2010b) with Image Processing toolbox (Version 7.1 -R2010b) using both known algorithms for image analysis and processing and those proposed by the author.

### Pre-processing

**Figure 2:**
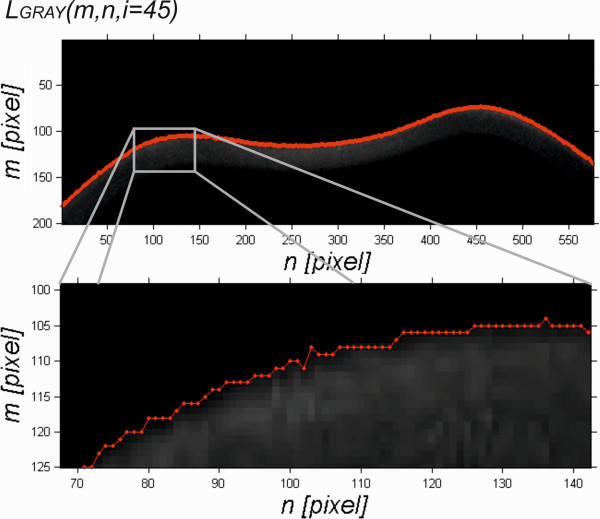
**Example of an input image** ***L***_***GRAY***_ **(** ***m,n,i*** **).** (where *m*-row *m*∈(1,*M*), *n*-column *n*∈(1,*N*), for *i* = 45 pixel) acquired from the Corvis tonometer (resolution *M* × *N* = 200 × 576 pixels) and the outer corneal contour *L*_*T*_(*n*,*i = 45*) (red).

The image pre-processing stage is partly known from previous publications of the author [52–54]. As mentioned earlier and in paper [52], the input images *L*_*GRAY*_(*m,n,i*) (where *m*-row *m*∈(1,*M*), *n*-column *n*∈(1,*N*), and *i* – another 2D image *i*∈(1,*I*)) acquired from the Corvis tonometer had an *M* × *N* × *I* resolution of 200 × 576 × 140 pixels - Figure 2. Pixels for this type of image matrix symbols are numbered from one, first rows (*m*) and then columns (*n*) and the image number in the sequence (*i*). As a result, corneal deformation is shown in the three-dimensional graph (Cartesian coordinate system) in the reverse form relative to the image *L*_*GRAY*_(*m,n,i*) visible in the Corvis tonometer. Input images were acquired directly in the *.cst format. First, a sequence of images *L*_*GRAY*_(*m,n,i*) underwent median filtering with a mask *h*_*1*_ sized *M*_*h1*_×*N*_*h1*_*×I*_*h1*_ = 3×3×3 pixels. The mask size was chosen arbitrarily, taking into consideration the size of artefacts and distortions that enter the optical path. Next, the filtered image *L*_*M*_(*m,n,i*) was subjected to further preliminary transformations. These transformations were developed and their repeatability and accuracy were corrected in previous papers [52, 54]. These include:

- detection of the outer edge of the cornea (using the Canny method [47]) - result *L*_*C*_(*m,n,i*),
- morphological close operation,
- corneal contour *L*_*T*_(*n*,*i*) (Figure 2),
- division of the contour *L*_*T*_(*n*,*i*) into the sum of components: *L*_*TD*_(*n*,*i*) – constant component of the corneal shape for *t* = 0 (for *i* = 1); *L*_*TR*_(*n*,*i*) – corneal deformation and *L*_*TO*_(*n*,*i*) – reaction of the eyeball (which is shown schematically in Figure 1).

**Figure 3:**
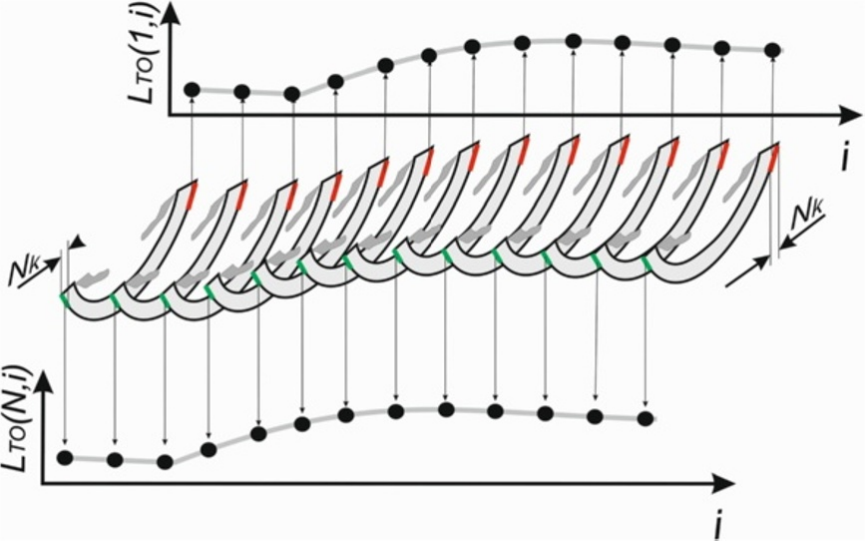
**Schematic diagram of the method for designating the boundary points of the reaction of the eyeball.** The waveforms *L*_*TO*_(*N,i*) and *L*_*TO*_(*1,i*) determined on this basis, shown demonstratively on the left and right side, are the basis for determining the missing values for *n*∈(2,3,…,*N*-2,*N*-1).

Designation of the waveform *L*_*TO*_(*n,i*) is possible owing to the analysis of the visible contour of the sclera at the border of the left and right image, which is shown schematically in Figure 3, i.e.:
12

**Figure 4:**
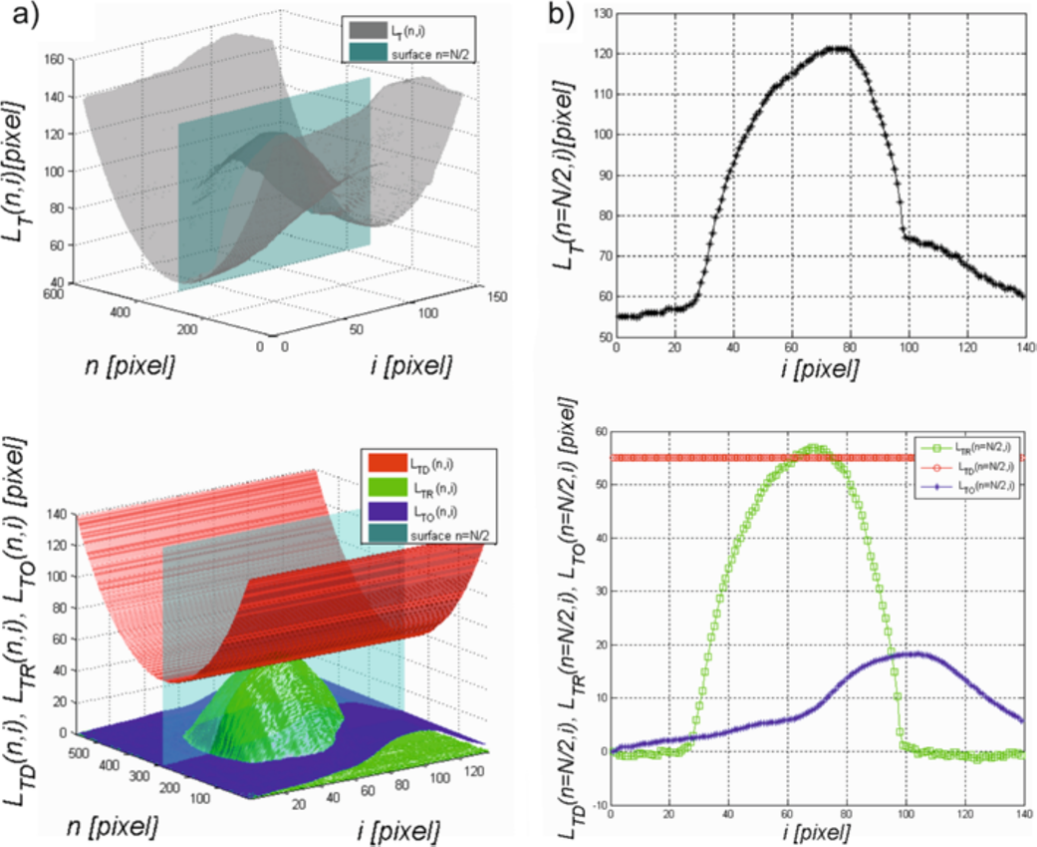
**Sample results obtained from the Corvis device.** The waveform *L*_*T*_(*n*,*i*) visible in figure **a)** is the response of the cornea and eyeball with a constant component to an air puff. On this basis and as a result of image pre-processing, *L*_*TD*_(*n*,*i*), *L*_*TR*_(*n*,*i*) and *L*_*TO*_(*n*,*i*) were automatically separated. Figure **b)** shows the results obtained for *n* = *N*/2 in the form of a two-dimensional graph.

The waveforms *L*_*TO*_(*N,i*) and *L*_*TO*_(*1,i*) designated based on the formulas (1) and (2) are the basis for determining the missing values for *n*∈(2,3,…,*N*-2,*N*-1). These values were determined based on linear interpolation. The results obtained, namely *L*_*TD*_(*n*,*i*), *L*_*TR*_(*n*,*i*), *L*_*TO*_(*n*,*i*) and the summary waveform *L*_*T*_(*n*,*i*), are shown in Figure 4. They are the basis for further processing steps presented hereafter.

### Processing

The results obtained, namely *L*_*TD*_(*n*,*i*), *L*_*TR*_(*n*,*i*), *L*_*TO*_(*n*,*i*) and the summary waveform *L*_*T*_(*n*,*i*), are subjected to further transformations, which allows for automatic determination of three new parameters:

- the degree of the corneal reaction relative to the static position,
- the corneal length changes,
- the ratio of amplitude changes to the corneal deformation length.

**Figure 5:**
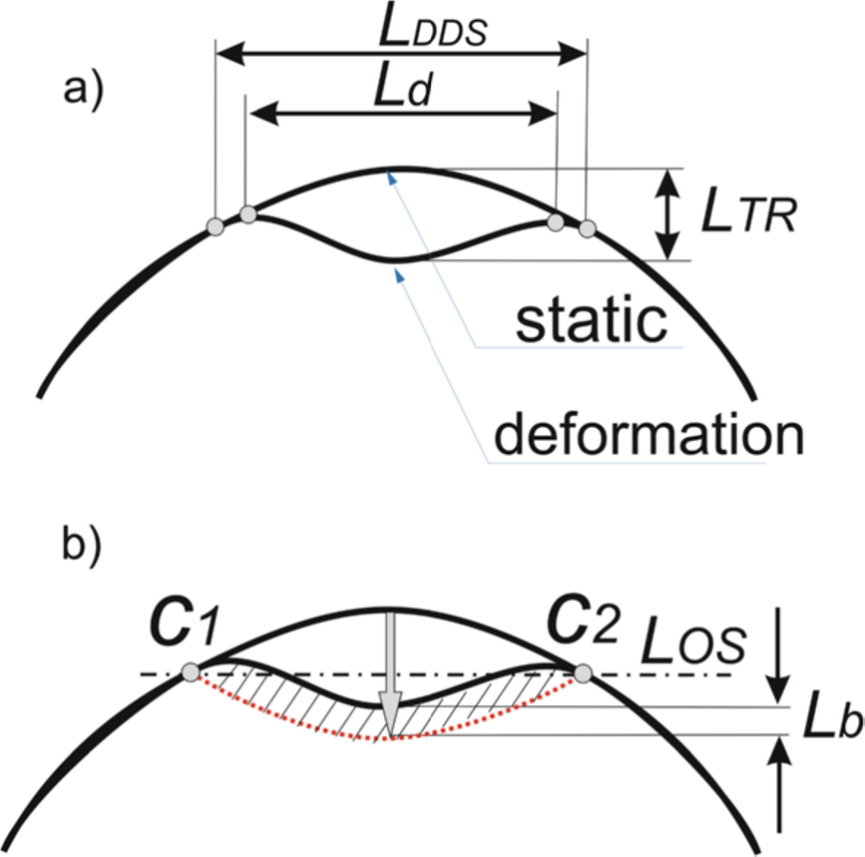
**Schematic diagram of the measurement method of the values** ***L***_***S***_ **(** ***n*** **,** ***i*** **) and** ***L***_***g***_ **(** ***n*** **,** ***i*** **).** Part **a)** shows the measurement of the distance *L*_*d*_ as pick distance and the distance *Ls* as the corneal length where deformation relative to the static position is measured. Part **b)** shows the measured deformation value *L*_*b*_ as a measure of the difference in relation to the original position.

**Determination of the degree of the corneal reaction relative to the static position** requires measurement of the length *L*_*S*_(*n*,*i*) and, on its basis, *L*_*g*_(*n*,*i*). The value *L*_*S*_(*n*,*i*) is the length of the cornea subjected to deformation. In contrast, *L*_*g*_(*n*,*i*) is the surface area of the difference between corneal deformation and its inverted static shape. The measurement method is shown schematically in Figure 5. The value *L*_*W*_(*n*,*i*) necessary for calculating *L*_*S*_(*n*,*i*) is determined on the basis of *L*_*TR*_(*n*,*i*), i.e.:
3

where *p*_*r*_ – binarization threshold set at the maximum possible amplitude of the noise, i.e. approximately two pixels [55].

Next, after median filtering of the waveform (*L*_*W*_(*n*,*i*)) with a filter mask *h* sized *M*_*h*_ × *N*_*h*_ = 3 × 3 pixels, the value *L*_*S*_(*n*,*i*) is obtained. In the next stage, a straight line *L*_*OS*_ (the axis of symmetry) is drawn in the range for which *L*_*S*_(*n*,*i*) = 1 for the extreme points *c*_*1*_ and *c*_*2*_ (Figure 5.) with the coordinates (*m*_*1*_*,n*_*1*_) and (*m*_*2*_*,n*_*2*_) respectively, i.e.:
4

Then each point of the corneal contour *L*_*T*_(*n*,*i*) is reflected symmetrically with respect to the axis of symmetry *L*_*OS*_(*n*,*i*) for which *L*_*S*_(*n*,*i*) = 1, which gives *L*_*K*_(*n*,*i*), i.e.:
5

**Figure 6:**
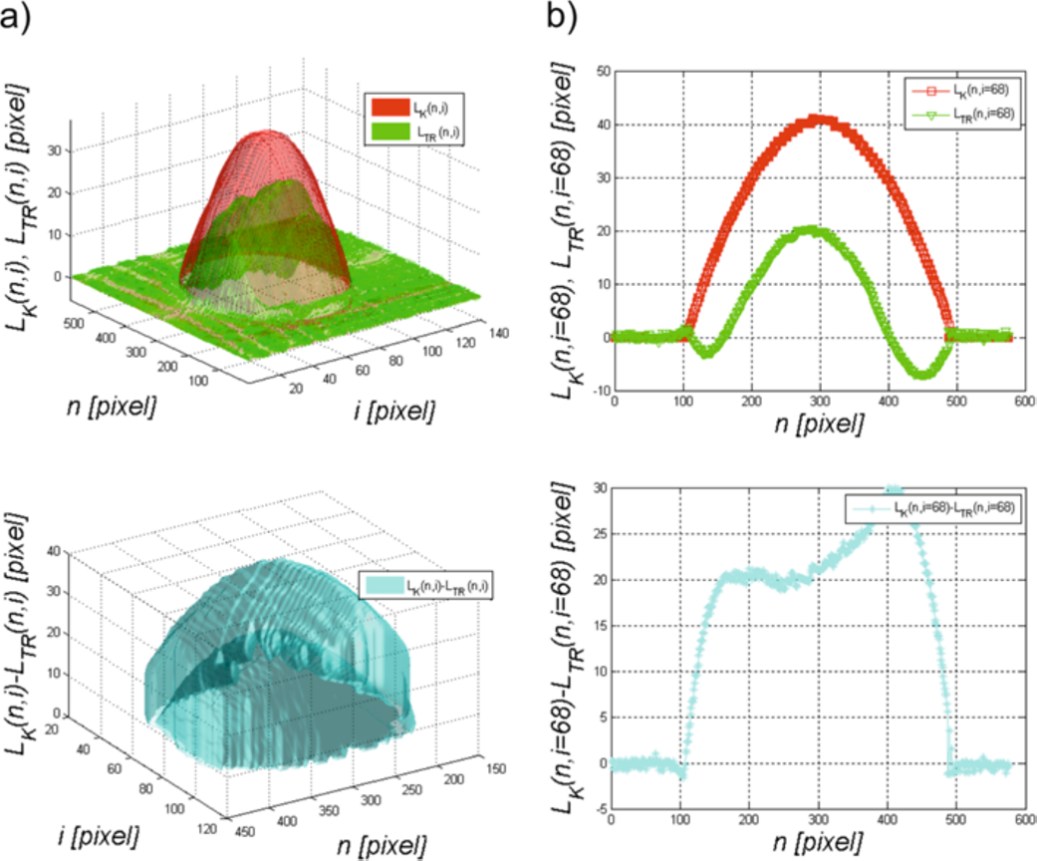
**Graphs** ***L***_***K***_ **(** ***n*** **,** ***i*** **) and** ***L***_***TR***_ **(** ***n*** **,** ***i*** **).** Part **a)** shows three-dimensional graphs *L*_*K*_(*n*,*i*) and *L*_*TR*_(*n*,*i*) and their difference, whereas part **b)** shows two-dimensional graphs for the sample value *i* = 68 pixels.

The result *L*_*K*_(*n*,*i*) is shown schematically with the dotted line in Figure 5.b). The results *L*_*K*_(*n*,*i*) and *L*_*TR*_(*n*,*i*), after the removal of the constant component (the static corneal contour *L*_*TD*_(*n*,*i*)), are shown in Figure 6.

**Determination of the corneal length changes** is the second calculated parameter and is directly linked to the results *L*_*K*_(*n*,*i*) and *L*_*TR*_(*n*,*i*). Changes in the corneal length are calculated within the range for which *L*_*S*_(*n*,*i*) = 1. Three values are calculated, the length of the corneal deformation *L*_*DDTR*_(*i*), the corneal length in a stable state covering the deformation area *L*_*DDK*_(*i*) and the distance between the extreme points of deformation *L*_*DDS*_(*i*) (Figure 5):
678

**Figure 7:**
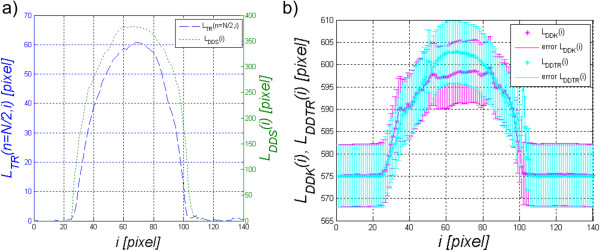
**Graph of distance between the extreme points of deformation** ***L***_***DDS***_ **(** ***n*** **,** ***i*** **) against the corneal reaction a), and a graph of corneal deformation length** ***L***_***DDTR***_ **(** ***n*** **,** ***i*** **) and the corneal length in a stable state covering the deformation area -** ***L***_***DDK***_ **(** ***n*** **,** ***i*** **) b).**

The obtained measurement results are shown in Figure 7a and b. Additionally, Figure 7b shows the range of measurement accuracy calculated as the sum of ± LSB (Least Significant Bit) for each distance between adjacent points. It results from high sensitivity of formulas (6) and (7) to noise, the sum of relationships *L*_*K*_(*n + 1*,*i*)-*L*_*K*_(*n*,*i*) and *L*_*TR*_(*n*,*i*)-*L*_*TR*_(*n*,*i*). It is therefore impossible at this stage to clearly assess whether the cornea changed its length during deformation. Accordingly, the approach was modified and waveforms *L*_*K*_(*n*,*i*) and *L*_*TR*_(*n*,*i*) were approximated with a polynomial of degree 8, thus obtaining *L*_*K2*_(*n*,*i*) and *L*_*TR2*_(*n*,*i*). The degree of the polynomial was chosen on the basis of the performed measurements of the best match. The corneal length measurement, analogously to equations (6) and (7), was marked as *L*_*AAK*_(*i*) and *L*_*AATR*_(*i*):
910

**Figure 8:**
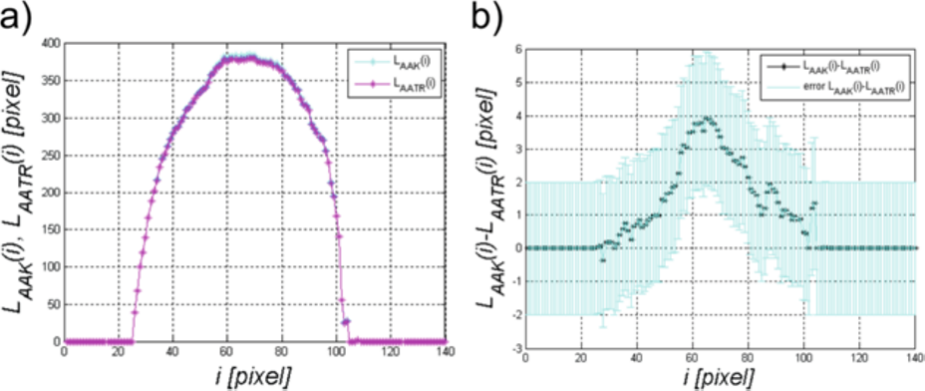
**Graph of corneal length during deformation and in a stable state** ***L***_***AAK***_ **(** ***i*** **) and** ***L***_***AATR***_ **(** ***i*** **) a) and the length difference** ***L***_***AAK***_ **(** ***i*** **)-** ***L***_***AATR***_ **(** ***i*** **) b).**

The sample results of changes in the values *L*_*AAK*_(*i*) and *L*_*AATR*_(*i*) are shown in Figure 8. The differences between the waveforms of corneal length changes *L*_*AAK*_(*i*) and *L*_*AATR*_(*i*) visible in Figure 8b) are in this case 4 ± 2 pixels.

### The ratio of the corneal deformation length to deformation amplitude

This parameter was previously calculated using equation (3) based on which the value *L*_*S*_(*n*,*i*) and then *L*_*DDS*_(*i*) are calculated (formula (8)). Thus, the ratio of the corneal deformation length to corneal deformation amplitude was calculated on the basis of *L*_*DDS*_(*i*) and *L*_*TR*_(*n,i*) as *L*_*DDS/TR*_(*i*), i.e.:
11

assuming .

**Figure 9:**
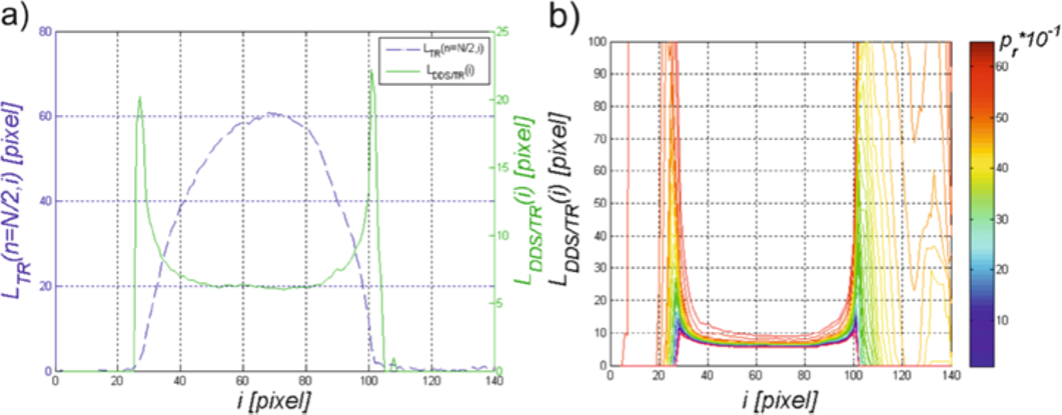
**Sample graph** ***L***_***DDS/TR***_ **(** ***i*** **).** The positions of the two peaks on the right and left side define the points of corneal applanation. Part **a)** shows the graph *L*_*DDS/TR*_(*i*) with the graph *L*_*TR*_(*i*) in the background, whereas part **b)** shows the impact of changes in the binarization threshold *p*_*r*_ on the measurement results obtained - the shape of the graph *L*_*DDS/TR*_(*i*).

**Figure 10:**
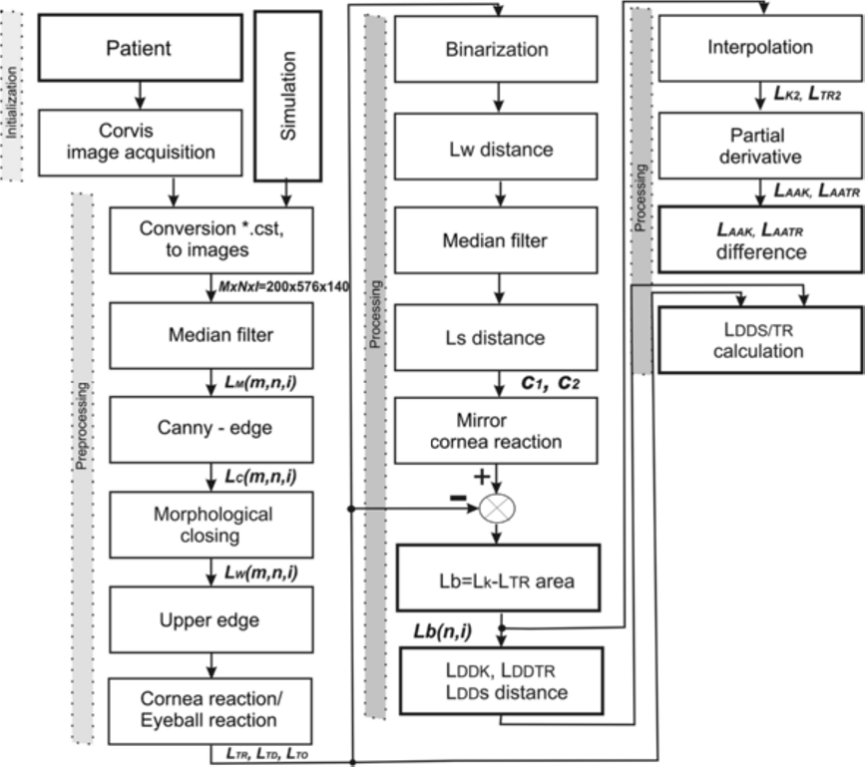
**Block diagram of the various processing stages.** In image pre-processing, data from the Corvis tonometer were analysed, which enabled to reconstruct changes in the shape of the cornea and separate the reaction of the eyeball. In the next stage of data processing, a detailed analysis of the response of the eyeball was performed.

A sample graph of *L*_*DDS/TR*_(*i*) is shown in Figure 9. The visible two peaks designate two points for which the corneal deformation length is greatest in relation to the deformation amplitude. The impact of changes in binarization threshold *p*_*r*_ (formula (3)) on the obtained results is shown in Figure 9b) for *p*_*r*_∈(0.1,6).The results obtained using the presented new methods of automated analysis are discussed in the next section. The individual processing steps, discussed above, are shown as a block diagram in Figure 10.

## Results

The proposed new algorithm allows for the automatic measurement of three new parameters of the corneal response during deformation using the Corvis tonometer. The calculation of the degree of the corneal reaction relative to the static position enabled automatic measurement of the difference between these waveforms, *L*_*K*_(*n,i* = 68) and *L*_*TR*_(*n,i* = 68), which was 30 pixels for *n* = 403 pixels (Figure 6). Based on the analysed 14000 2D images, the corneal reaction was always lower compared to the corresponding static position. For all the analysed images *L*_*K*_(*n,i* = 68)-*L*_*TR*_(*n,i* = 68) > 0 in each case. Due to the high sensitivity of the method (formulas (6)(7)), the analysis of changes in the corneal length *L*_*DDK*_(*i*) and *L*_*DDTR*_(*i*) must be preceded by filtering or interpolation. The differences between the waveforms of changes in the corneal length *L*_*AAK*_(*i*) and *L*_*AATR*_(*i*) visible in Figure 8b) are in this case 4 ± 2 pixels (about 1% in relation to its original length). These differences are different for various groups of images and on average they are a few pixels. In the analysis of this group of results, the measurement error and accuracy of obtaining the results *L*_*K2*_(*i*), *L*_*TR2*_(*i*) and, in particular, *L*_*T*_(*n*,*i*), should be taken into account. Errors may arise in the initial phase of the algorithm operation in the form of not fully visible corneal contours. They replicate here introducing a substantial measurement error [56]. They also affect the results obtained from the calculation of the third parameter – the ratio of deformation to the corneal deformation length. This parameter designates two peaks (Figure 9). From a practical point of view, the position of the two peaks and the shape of the graph *L*_*DDS/TR*_(*i*) between them are important. This fragment on the graph (Figure 9) runs stably for the approximate value of *i*∈(50,85). It means that the ratio of the deformation to deformation amplitude is constant. The cornea during deformation causes proportional changes in the deformation length. The position of the peaks (*a*_*1*_, *a*_*2*_) is slightly dependent on the accuracy of the earlier analysis and especially on the adopted binarization threshold *p*_*r*_. Tab. 1 shows the obtained results of changes in the position (relative to i) of the two peaks (a_1_, a_2_), for the adopted different binarization thresholds, i.e. for *p*_*r*_∈(0.1,6). According to the presented results, changes in peak positions are at a level of the resolution error (±1 pixel) for small values of the threshold *p*_*r*_. In a further step, the correlation between the position of the peaks and applanation points, calculated in the standard software of the Corvis tonometer, was assessed. This correlation was 92%, which confirms the usefulness of the new discussed method for the automatic designation of applanation points. Typical analysis of applanation points may also be carried out in a conventional manner as a search for time instants (values *i*) for which corneal flattening occurs. This is a difficult task in terms of algorithmics due to the need to correct the angle of the cornea position relative to the tonometer or difficulties in the unambiguous determination of corneal flattening areas. Particular attention should be paid here to the calculation of the above errors that apply only to the discussed method. In practice, especially when calculating their values for a different type of a tonometer, for a different type of Scheimpflug camera, close attention should be paid to the accuracy and reproducibility of obtaining images *L*_*GRAY*_(*m,n,i*). This also applies to data derived from simulation as well as, for example, data from the measured eye phantom. Diversity of technological parameters of the Corvis tonometer and the impact of other factors on the result and reproducibility of measurements should be taken into account: e.g. the impact of the patient’s head position or the effect of temperature and humidity. In extreme cases, these errors can skew each other or greatly increase the total measurement error.

**Table 1:** **Results of position changes (relative to** ***i*** **) of two peaks (applanation points) for the adopted different binarization thresholds,** ***p***_***r***_ **∈(0.1,6)**

***p***_***r***_	0.1	0.5	1	1.5	2	2.5	3	3.5	4	4.5	5	5.5	6
***a***_***1***_ **[pixel]**	22	25	26	27	27	27	27	28	29	28	29	29	30
***a***_***2***_ **[pixel]**	119	107	102	101	101	101	101	101	101	101	100	100	100

The presented new features enable to extend the range of possibilities for quantitative assessment of the corneal response and to find links with known features measured in the Corvis tonometer. The proposed algorithm provides reproducible results fully automatically using Core i7 10GB RAM within a few seconds per patient.

### Critical summary

The paper presents a method for calculating three additional parameters of the quantitative assessment of corneal deformation. The presented algorithm for image analysis provides reproducible results in a fully automated manner. The presented algorithm is only one of several possible solutions to this task. In particular, the described profiled algorithm can be composed of other image analysis and processing methods [56]. For example, the presented problem of image analysis and processing can be solved using other methods [57, 58] derived from morphological transformations (open, close etc.) [59–61]. However, in any case, the algorithm must be profiled to a given application. Proper selection of the algorithm structure is an important part taking into account, on the one hand, full automation, and, on the other hand, large variation in images (both their quality as well as the parameters of the cornea itself). Due to full automation of the measurement, there is a need to profile the algorithm for a particular research problem and a group of images. On the other hand, the algorithm requires generalization in order to ensure its correct operation in different research institutions. Its final form is thus a compromise between these two elements.

For example, in paper [52], the authors present the division of corneal response into four different groups depending on the characteristics of their response to an air puff. New parameters that are not calculated in the Corvis tonometer are introduced there. For example, in papers [47, 62–65], conclusions and the whole analysis are only related to the parameters available from the Corvis tonometer. Paper [54] shows the correlation of the new parameters obtained from the Corvis tonometer with known parameters available in the proprietary software of the Corvis tonometer.

In subsequent papers, the author intends to:

- verify the repeatability of the three parameters obtained for the same patients measured with different types of the Corvis tonometer at different medical centres [56, 66],
- perform quantitative analysis of the impact of patient positioning on the results obtained [66].

Biomechanical parameters of the eye, measured in dynamic states, still represent a new area of interdisciplinary research combining engineering, medicine and computer science [67, 68].

## Abbreviations

ORA: Ocular Response Analyzer.

## References

[1] Śródka W (2013). Applanation pressure function in Goldmann tonometry and its correction. Acta Bioeng Biomech.

[2] Śródka W (2011). Evaluating the material parameters of the human cornea in a numerical model. Acta Bioeng Biomech.

[3] Smedowski A, Weglarz B, Tarnawska D, Kaarniranta K, Wylegala E (2014). Comparison of three intraocular pressure measurement methods including biomechanical properties of the cornea. Invest Ophthalmol Vis Sci.

[4] Shah S, Laiquzzaman M, Mantry S, Cunliffe I (2008). Ocular response analyzer to assess hysteresis and corneal resistance factor in low tension, open angle glaucoma and ocular hypertension. Clin Experiment Ophthalmol.

[5] Hallahan KM, Sinha Roy A, Ambrosio R, Salomao M, Dupps WJ (2014). Discriminant value of custom ocular response analyzer waveform derivatives in keratoconus. Ophthalmology.

[6] Wells AP, Garway-Heath DF, Poostchi A, Wong T, Chan KCY, Sachdev N (2008). Corneal hysteresis but not corneal thickness correlates with optic nerve surface compliance in glaucoma patients. Invest Ophthalmol Vis Sci.

[7] Luce DA (2005). Determining in vivo biomechanical properties of the cornea with an ocular response analyzer. J Cataract Refract Surg.

[8] Malik NS, Moss SJ, Ahmed N, Furth AJ, Wall RS, Meek KM (1992). Ageing of the human corneal stroma: structural and biochemical changes. Biochim Biophys Acta.

[9] Sherrard ES, Novakovic P, Speedwell L (1987). Age-related changes of the corneal endothelium and stroma as seen in vivo by specular microscopy. Eye (Lond).

[10] Kotecha A, Elsheikh A, Roberts CR, Zhu HG, Garway-Heath DF (2006). Corneal thicknessand age-related biomechanical properties of the cornea measured with the ocular response analyzer. Invest Ophthalmol Vis Sci.

[11] Lopes B, Ramos I, Ambrósio R (2014). Corneal densitometry in Keratoconus. Cornea.

[12] Valbon BF, Ambrósio-Jr R, Fontes BM, Alves MR (2013). Effects of age on corneal deformation by non-contact tonometry integrated with an ultra-high-speed (UHS) Scheimpflug camera. Arq Bras Oftalmol.

[13] Brown KE, Congdon NG (2006). Corneal structure and biomechanics: impact on the diagnosis and management of glaucoma. Curr Opin Ophthalmol.

[14] Ambrósio R, Valbon BF, Faria-Correia F, Ramos I, Luz A (2013). Scheimpflug imaging for laser refractive surgery. Curr Opin Ophthalmol.

[15] Ozer MA, Acar M, Yildirim C (2014). Intraocular pressure-lowering effects of commonly used fixed combination drugs with timolol in the management of primary open angle glaucoma. Int J Ophthalmol.

[16] Borrego SL, Morales L, Martínez de-la-Casa JM, Sáenz-Francés F, Fuentes M, Feijóo JG (2014). The Icare-Pro Rebound Tonometer Versus the Hand-held Applanation Tonometer in Congenital Glaucoma. J Glaucoma.

[17] Salvetat ML, Zeppieri M, Tosoni C, Felletti M, Grasso L, Brusini P (2014). Corneal Deformation Parameters Provided by the Corvis-ST Pachy-Tonometer in Healthy Subjects and Glaucoma Patients. J Glaucoma.

[18] Shin J, Lee JW, Kim EA, Caprioli J (2014). The Effect of Corneal Biomechanical Properties on Rebound Tonometer in Patients with Normal Tension Glaucoma. Am J Ophthalmol.

[19] Dupps WJ, Wilson SE (2006). Biomechanics and wound healing in the cornea. Exp Eye Res.

[20] Elsheikh A, Alhasso D, Rama P (2008). Assessment of the epithelium’s contribution to corneal biomechanics. Exp Eye Res.

[21] Kling S, Bekesi N, Dorronsoro C, Pascual D, Marcos S (2014). Corneal viscoelastic properties from finite-element analysis of in vivo air-puff deformation. PLoS ONE.

[22] Congdon NG, Broman AT, Bandeen-Roche K, Grover D, Quigley HA (2006). Central corneal thickness and corneal hysteresis associated with glaucoma damage. Am J Ophthalmol.

[23] Touboul D, Roberts C, Kerautret J, Garra C, Maurice-Tison S, Saubusse E, Colin J (2008). Correlation between corneal hysteresis intraocular pressure, and corneal central pachymetry. J Cataract Refract Surg.

[24] Huseynova T, Waring GO, Roberts C, Krueger RR, Tomita M (2014). Corneal biomechanics as a function of intraocular pressure and pachymetry by dynamic infrared signal and Scheimpflug imaging analysis in normal eyes. Am J Ophthalmol.

[25] Gatinel D, Chaabouni S, Adam PA, Munck J, Puech M, Hoang-Xuan T (2007). Corneal hysteresis, resistance factor, topography, and pachymetry fter corneal lamellar flap. J Refract Surg.

[26] Kirwan C, O’Keefe M, Lanigan B (2006). Corneal hysteresis and intraocular pressure measurement in children using the Reichert ocular response analyzer. Am J Ophthalmol.

[27] Shah S, Laiquzzaman M, Cunliffe I, Mantry S (2006). The use of the Reichert ocular response analyser to establish the relationship between ocular hysteresis, corneal resistance factor and central corneal thickness in normal eyes. Cont Lens Anterior Eye.

[28] Tao C, Han Z, Sun Y, Zhou C, Roberts C, Zhou D, Ren Q (2013). Corneal hysteresis with intraocular pressure of a wide range: a test on porcine eyes. J Refract Surg.

[29] Anand A, De Moraes CGV, Teng CC, Tello C, Liebmann JM, Ritch R (2010). Lower corneal hysteresis predicts laterality in asymmetric open angle glaucoma. Invest Ophthalmol Vis Sci.

[30] Marjanović I, Martinez A, Marjanović M, Milić N, Kontić D, Hentova-Senćanić P, Marković V, Bozić M (2014). Changes in the retrobulbar hemodynamic parameters after decreasing the elevated intraocular pressure in primary open-angle glaucoma patients. Srp Arh Celok Lek.

[31] Ortiz D, Pinero D, Shabayek MH, Arnalich-Montiel F, Alió JL (2007). Corneal biomechanical properties in normal, post-laser in situ keratomileusis, and keratoconic eyes. J Cataract Refract Surg.

[32] Shah S, Laiquzzaman M, Bhojwani R, Mantry S, Cunliffe I (2007). Assessment of the biomechanical properties of the cornea with the ocular response analyzer in normal and keratoconic eyes. Invest Ophthalmol Vis Sci.

[33] Bak-Nielsen S, Pedersen IB, Ivarsen A, Hjortdal J (2014). Dynamic Scheimpflug-based assessment of keratoconus and the effects of corneal cross-linking. J Refract Surg.

[34] Pepose JS, Feigenbaum SK, Qazi MA, Sanderson JP, Roberts CJ (2007). Changes in corneal biomechanics and intraocular pressure following LASIK using static, dynamic, and noncontact tonometry. Am J Ophthalmol.

[35] Pedersen IB, Bak-Nielsen S, Vestergaard AH, Ivarsen A, Hjortdal J (2014). Corneal biomechanical properties after LASIK, ReLEx flex, and ReLEx smile by Scheimpflug-based dynamic tonometry. Graefes Arch Clin Exp Ophthalmol.

[36] Meek KM, Leonard DW (1993). Ultrastructure of the corneal stroma: a comparative study. Biophys J.

[37] Maeda N, Ueki R, Fuchihata M, Fujimoto H, Koh S, Nishida K (2014). Corneal biomechanical properties in 3 corneal transplantation techniques with a dynamic Scheimpflug analyzer. Jpn J Ophthalmol.

[38] Correia FF, Ramos I, Roberts CJ, Steinmueller A, Krug M, Ambrósio R (2013). Impact of chamber pressure on the deformation response of corneal models measured by dynamic ultra-high-speed Scheimpflug imaging. Arq Bras Oftalmol.

[39] Kotecha A (2007). What biomechanical properties of the cornea are relevant for the clinician?. Surv Ophthalmol.

[40] Ambrósio R, Ramos I, Luz A, Faria F, Steinmueller A, Krug M, Belin M, Roberts CJ (2013). Dynamic ultra high speed Scheimpflug imaging for assessing corneal biomechanical properties. Revista Brasileira de Oftalmologia.

[41] Elsheikh A, Anderson K (2005). Comparative study of corneal strip extensometry and inflation tests. J R Soc Interface.

[42] Papastergiou GI, Kozobolis V, Siganos DS (2010). Effect of recipient corneal pathology on Pascal tonometer and Goldmann tonometer readings in eyes after penetrating keratoplasty. Eur J Ophthalmol.

[43] Herdener S, Hafizovic D, Pache M, Lautebach S, Funk J (2008). Is the PASCAL-Tonometer suitable for measuring intraocular pressure in clinical routine? Long- and short-term reproducibility of dynamic contour tonometry. Eur J Ophthalmol.

[44] Fontes BM, Ambrosio R, Alonso RS, Jardim D, Velarde GC, Nose W (2008). Corneal biomechanical metrics in eyes with refraction of -19.00 to +9.00 D in healthy brazilian patients. J Refract Surg.

[45] Sullivan-Mee M, Billingsley S, Patel AD, Halverson KD, Alldredge BR, Qualls C (2008). Ocular Response Analyzer in subjects with and without Glaucoma. Optom Vis Sci.

[46] Pillunat LE, Anderson DR, Knighto N, Joos KM, Feuer WJ (1997). Autoregulation of human optic nerve in response to increased intraocular pressure. Exp Eye Re.

[47] Sawada A, Yamada H, Yamamoto Y, Yamamoto T (2014). Intraocular pressure alterations after visual field testing. Jpn J Ophthalmol.

[48] Metzler KM, Mahmoud AM, Liu J, Roberts CJ (2014). Deformation response of paired donor corneas to an air puff: intact whole globe versus mounted corneoscleral rim. J Cataract Refract Surg.

[49] Han Z, Tao C, Zhou D, Sun Y, Zhou C, Ren Q, Roberts CJ (2014). Air puff induced corneal vibrations: theoretical simulations and clinical observations. J Refract Surg.

[50] Mastropasqua L, Lanzini M, Curcio C, Calienno R, Mastropasqua R, Colasante M, Mastropasqua A, Nubile M (2014). Structural modifications and tissue response after standard epi-off and iontophoretic corneal crosslinking with different irradiation procedures. Invest Ophthalmol Vis Sci.

[51] Tejwani S, Shetty R, Kurien M, Dinakaran S, Ghosh A, Roy AS (2014). Biomechanics of the Cornea Evaluated by Spectral Analysis of Waveforms from Ocular Response Analyzer and Corvis-ST. PLoS ONE.

[52] Koprowski R, Lyssek-Boron A, Nowinska A, Wylegala E, Kasprzak H, Wrobel Z (2014). Selected parameters of the corneal deformation in the Corvis tonometer. Biomed Eng Online.

[53] Koprowski R, Kasprzak H, Wróbel Z (2014). New automatic method for analysis and correction of image data from the Corvis tonometer. Comput Methods Biomech Biomed Engin.

[54] Koprowski R, Wilczyński S, Nowinska A, Lyssek-Boron A, Teper S, Wylegala E, Wróbel Z (2014). Quantitative assessment of responses of the eyeball based on data from the Corvis tonometer. Comput Biol Med.

[55] Otsu N (1979). A threshold selection method from gray-level histograms. IEEE Trans Sys Man Cyber.

[56] Koprowski R (2014). Quantitative assessment of the impact of biomedical image acquisition on the results obtained from image analysis and processing. Biomed Eng Online.

[57] Koprowski R, Wrobel Z (2008). Identification of layers in a tomographic image of an eye based on the canny edge detection. Inf Technol Biomed Adv Intell Soft Comput.

[58] Koprowski R, Wróbel Z (2009). Layers recognition in tomographic eye image based on random contour analysis. Computer recognition systems 3. Adv Intell Soft Comput.

[59] Jaworek-Korjakowska J, Tadeusiewicz R (2013). Assessment of dots and globules in dermoscopic color images as One of the 7-point check list criteria. The International Conference on Image Processing.

[60] Korzynska A, Iwanowski M (2012). Multistage morphological segmentation of bright-field and fluorescent microscopy images. Opt-Electron Rev.

[61] Jaworek-Korjakowska J, Tadeusiewicz R (2013). Hair removal from dermoscopic color images. Bio Algorithm Med Syst.

[62] Shen Y, Chen Z, Knorz MC, Li M, Zhao J, Zhou X (2014). Comparison of corneal deformation parameters after SMILE, LASEK, and femtosecond laser-assisted LASIK. J Refract Surg.

[63] Ali NQ, Patel DV, McGhee CN (2014). Biomechanical responses of healthy and keratoconic corneas measured using a noncontact scheimpflug-based tonometer. Invest Ophthalmol Vis Sci.

[64] Bañeros-Rojas P, de la Casa JM M, Arribas-Pardo P, Berrozpe-Villabona C, Toro-Utrera P, García-Feijoó J (2014). Comparison between Goldmann, Icare Pro and Corvis ST tonometry. Arch Soc Esp Oftalmol.

[65] Tian L, Huang YF, Wang LQ, Bai H, Wang Q, Jiang JJ, Wu Y, Gao M (2014). Corneal biomechanical assessment using corneal visualization scheimpflug technology in keratoconic and normal eyes. J Ophthalmol.

[66] Foster KR, Koprowski R, Skufca JD (2014). Machine learning, medical diagnosis, and biomedical engineering research - commentary. Biomed Eng Online.

[67] Wang S, Larin KV (2014). Shear wave imaging optical coherence tomography (SWI-OCT) for ocular tissue biomechanics. Opt Lett.

[68] Tao A, Chen Z, Shao Y, Wang J, Zhao Y, Lu P, Lu F (2013). Phacoemulsification induced transient swelling of corneal Descemet’s Endothelium Complex imaged with ultra-high resolution optical coherence tomography. PLoS ONE.

